# Deep learning-based landslide susceptibility mapping

**DOI:** 10.1038/s41598-021-03585-1

**Published:** 2021-12-16

**Authors:** Mohammad Azarafza, Mehdi Azarafza, Haluk Akgün, Peter M. Atkinson, Reza Derakhshani

**Affiliations:** 1 Department of Civil Engineering, University of Tabriz, Tabriz, Iran; 2 Department of Computer Engineering, University of Tabriz, Tabriz, Iran; 3 Department of Geological Engineering, Middle East Technical University (METU), Ankara, Turkey; 4 Lancaster Environment Centre, Faculty of Science and Technology, Lancaster University, Lancaster, UK; 5 Department of Geology, Shahid Bahonar University of Kerman, Kerman, Iran; 6 Department of Earth Sciences, Utrecht University, Utrecht, The Netherlands

**Keywords:** Natural hazards, Solid Earth sciences

## Abstract

Landslides are considered as one of the most devastating natural hazards in Iran, causing extensive damage and loss of life. Landslide susceptibility maps for landslide prone areas can be used to plan for and mitigate the consequences of catastrophic landsliding events. Here, we developed a deep convolutional neural network (CNN–DNN) for mapping landslide susceptibility, and evaluated it on the Isfahan province, Iran, which has not previously been assessed on such a scale. The proposed model was trained and validated using training (80%) and testing (20%) datasets, each containing relevant data on historical landslides, field records and remote sensing images, and a range of geomorphological, geological, environmental and human activity factors as covariates. The CNN–DNN model prediction accuracy was tested using a wide range of statistics from the confusion matrix and error indices from the receiver operating characteristic (ROC) curve. The CNN–DNN model was evaluated comprehensively by comparing it to several state-of-the-art benchmark machine learning techniques including the support vector machine (SVM), logistic regression (LR), Gaussian naïve Bayes (GNB), multilayer perceptron (MLP), Bernoulli Naïve Bayes (BNB) and decision tree (DT) classifiers. The CNN–DNN model for landslide susceptibility mapping was found to predict more accurately than the benchmark algorithms, with an AUC = 90.9%, IRs = 84.8%, MSE = 0.17, RMSE = 0.40, and MAPE = 0.42. The map provided by the CNN–DNN clearly revealed a high-susceptibility area in the west and southwest, related to the main Zagros trend in the province. These findings can be of great utility for landslide risk management and land use planning in the Isfahan province.

## Introduction

Landslides, one of the most common and potentially catastrophic geo-hazards, are complicated geological phenomena that occur in many geospatial environments and geomaterials^1–5^. Landslides are considered the second largest geo-hazard globally, causing extensive financial losses annually, according to the United Nations Development Program^6–8^. Current opinion is that the best way to minimise landslide risk is to monitor, assess and pinpoint landslide-prone areas reliably^9^. Thus, mapping landslide-susceptible areas can be essential to manage and restrict the potential impacts of landslides in vulnerable regions^10–17^. Landslide susceptibility assessment is not straightforward and generally requires detailed investigation of a range of factors underpinning susceptibility to produce zonation maps which delineate susceptible regions in a spatially explicit manner. Such spatial information on susceptibility can be especially valuable in policy-making and management decision-making to mitigate and reduce the risks related to landsliding^18–21^.

Landslide susceptibility mapping has been undertaken based on quantitative, semi-quantitative and qualitative methods (which can be further categorised as deterministic, statistic-probabilistic, heuristic, inventory-based, geostatistical and knowledge-based)^22–32^. Residual uncertainty within landslide susceptibility assessments has led to the development of more complex approaches to attain acceptable levels of accuracy. The largest sources of uncertainty in susceptibility modelling are related to the inventory database. Geological complexity, geomorphological deformations and land-use and landscape changes are the main causes of the uncertainties^33^. In this regard, development of more accurate models is important. Recently, knowledge-based approaches, namely, machine learning techniques such as logistic regression, support vector machines, random forests, artificial neural networks, and deep neural networks, have been applied for landslide susceptibility mapping to increase mapping accuracy^22,34–36^. These methods have improved capabilities concerning process adaptability and precision^37,38^.

**Figure 1:**
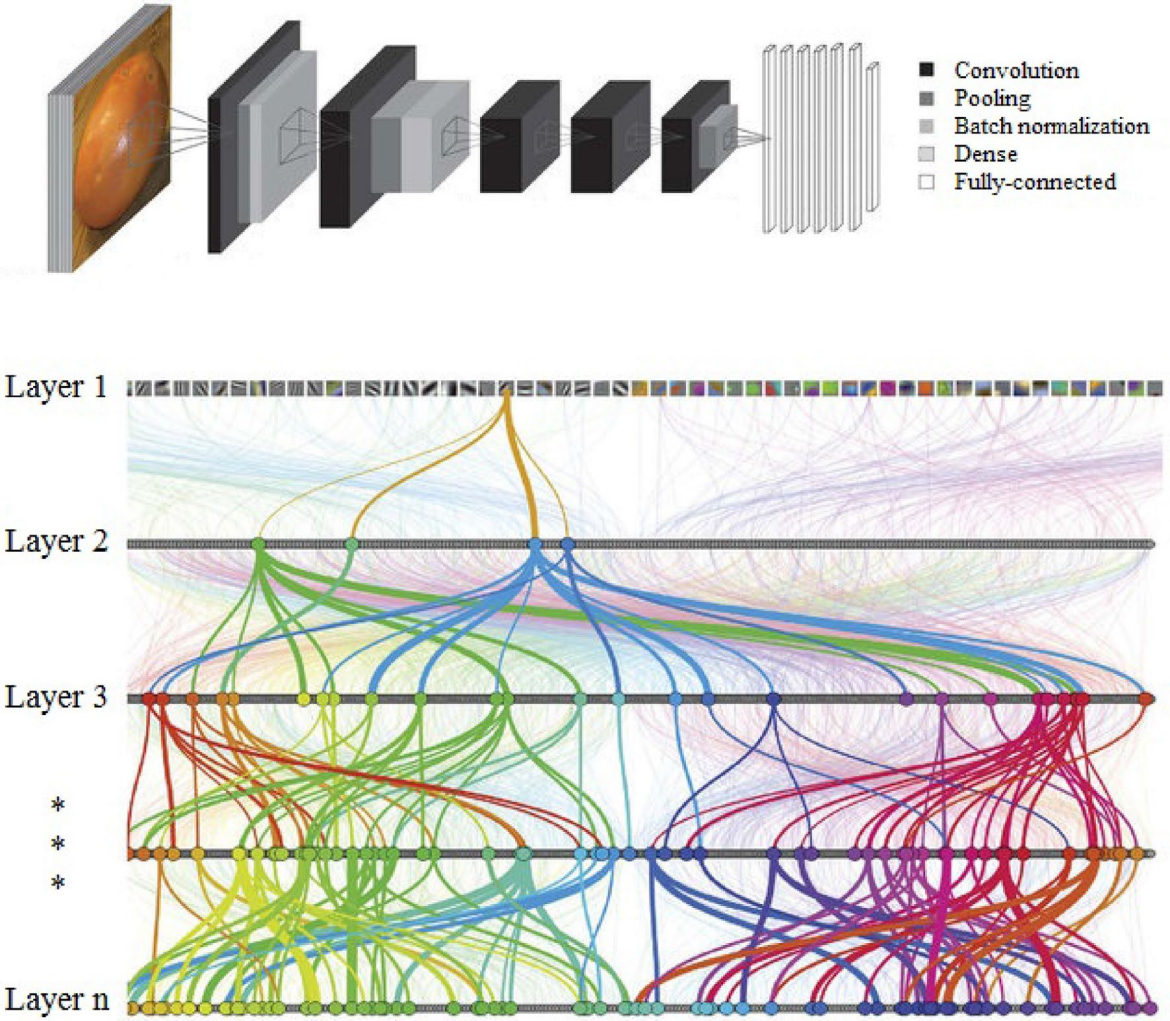
The main CNN–DNN architecture^48^.

Shallow learning (e.g., the multilayer perceptron, MLP) is machine learning where the learning is from data described by pre-defined (i.e., manually extracted) features. In deep learning, the feature extraction is computed automatically without manual human intervention. Deep learning methods have gained popularity because they often outperform conventional shallow learning methods by extracting informative features automatically from raw data with little or no pre-processing due to their complex architecture^39,40^. Deep learning networks (DNNs) have become extremely popular, including convolutional neural networks (CNNs). The CNN is a regularised version of a supervised learning framework that employs a sequence of mathematical operations arranged in network layers, including the convolution, pooling, batch normalisation, dense, dropout and fully-connected layers^41^. The general CNN architecture is illustrated in Fig. 1. CNNs are used commonly for object detection, feature extraction, pattern recognition, and land-cover exploration. These deep learning techniques are applied mostly to analyse remote sensing images with special emphasis on detecting and recognising 'events' and pattern recognition problems^42^. In terms of landslide susceptibility, CNNs are suitable for detecting historical landslide locations and landslide hazard analysis^8^. CNNs have also been applied for landslide recognition based on remote sensing images^32,37,42,43^. In relation to landslide susceptibility assessment, research has shown that deep learning is more effective than shallow learning^44–47^. While the application of CNNs has providedd increased accuracy for landslide susceptibility mapping, it has not provided the desirable or consistent accuracy. Thus, hybrid models are considered in this research.

This research aimed to assess the suitability of a *coupled* CNN–DNN neural network for landslide susceptibility analysis. The assessment was undertaken in the Isfahan province, Iran. The folowing objectives were set: (*i*) what are the main triggering factors for landslide occurrence probability?, (*ii*) Can the CNN–DNN predictive model provide more accurate results than regular models? and (*iii*) Can the CNN–DNN model provide the highest accuracy for susceptibility mapping. The CNN–DNN model was evaluated against a series of benchmark machine learning techniques, including the support vector machine (SVM), logistic regression (LR), Gaussian naïve Bayes (GNB), multilayer perceptron (MLP), Bernoulli naïve Bayes (BNB) and decision tree (DT) classifiers. After preparing the landslide 'covariates' (or factors) relevant to landslide occurrence in the study area, the various algorithms were used to predict landslide susceptibility spatially, and areas of high susceptibility were investigated further. The prediction results were tested using confusion matrices (i.e., overall accuracy, precision, recall and F1-score) and receiver operating characteristic curves (ROC).

## Analysis method

In deep learning and data mining, the extraction of features plays an important role. These extracted features can be used for classification or prediction with high accuracy. Since spatial prediction (i.e., mapping) is crucial for a range of applications including crisis management, urban planning and geo-hazard assessment (including landslide susceptibility assessment), the coupled CNN and DNN classifier has found wide applicability^8,37,42,49^. In the CNN–DNN classifier, the input data are evaluated by convolution, pooling, batch normalisation, dense, dropout and fully connected layers to predict the outputs (Fig. 1). The number of layers can be increased, thus, increasing the learning depth. The input data provide the first layer of evaluation as a data matrix in which each element has a specific feature value. Hence, the input layer is the primary feature map modified and organised by each convolutional layer and unit. These units extract different features from the input data. The first convolutional layer extracts some low-level features (e.g., lines, edges, corners). Further convolutional layers learn iteratively more intricate representations or features. Pooling is a critical manipulation in a CNN. Max-pooling is the most common manipulation amongst the different pooling approaches. Max-pooling aims to divide the feature maps into several rectangular zones and provide the maximum value for each zone^42^. Batch normalisation (or *batch*-norm) aims to increase the speed, performance and stability of the network. Batch normalisation is used to normalise the input layer by re-centring and re-scaling. The dense or regular densely-connected layer is commonly used as a linear/non-linear layer applied to the input and returned to the output. Fully connected layers connect every neuron in a preceding layer to every neuron in a subsequent layer. This is, in principle, the same as the traditional MLP network^41^. Combining these layers in the sequence can extract the desired features and, thereby, classify the input data into the desired classes.

Knowledge-based approaches have received significant attention in landslide susceptibility analyses where machine learning methods such as the CNN and DNN have provided highly accurate results. These methods, now considered common procedures, are applied to analyse visual imagery for image recognition and classification. CNNs and DNNs are regularised versions of the MLP, consisting of an input and an output layer and multiple hidden layers. The hidden layers of the CNN are typically concluded with a series of convolutional layers with multiplication or another dot product (e.g., the activation function is mostly RELU). On the other hand, the DNN finds the correct mathematical manipulation to transform the input into the output (based on linear or non-linear relationships). Each mathematical manipulation is considered a layer, and complex DNNs have many layers^40,41^. Since 2019, the application of the CNNs and DNNs in landslide susceptibility analyses has led to establishment of the potential of deep learning for landslide susceptibility mapping^32,42,46^. More widely, implementation of the coupled CNN–DNN has led to increased accuracy compared to the implementation of these two methods separately. We, thus, develop a coupled CNN–DNN methodology to assess landslide susceptibility.

### Study site and data

#### Study location

**Figure 2:**
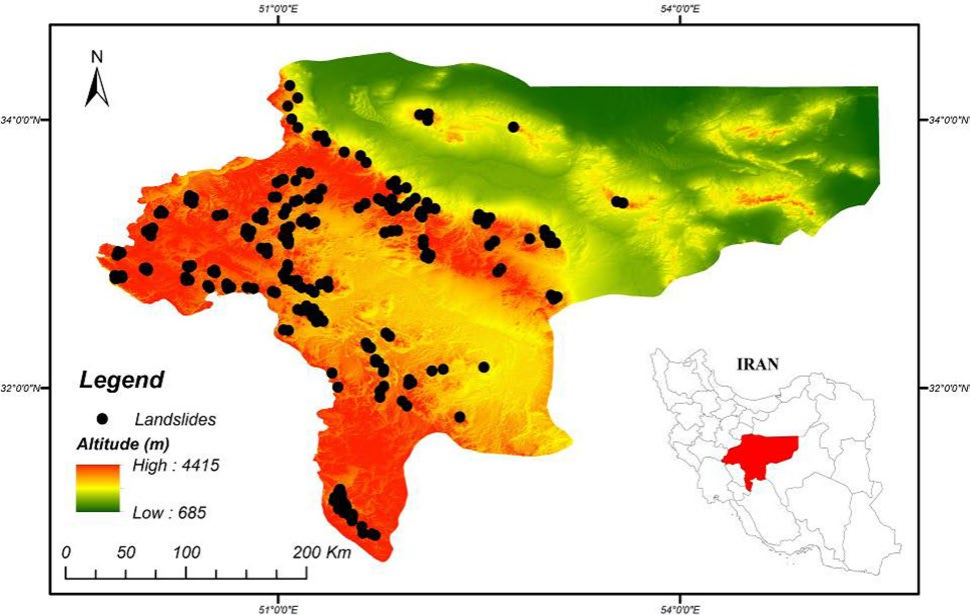
Location map of the study region using ArcGIS 10.4.1 software package^55^.

The study area is located in the Isfahan province of central Iran and covers an area of approximately 106,786 km^2^ (Fig. 2). Markazi, Qom and Semnan provinces are located to the north, and the Fars and Kohgiluyeh-Boyerahmad provinces are located to the south of the Isfahan province. The city of Isfahan, which is the capital of the Isfahan province, is considered to be the historical, cultural and touristic capital of Iran. The Isfahan province experiences a moderate and dry climate that ranges from 10.6 to 40.6 °C annually (the average annual temperature has been recorded as 16.7 °C). The annual rainfall of Isfahan has been recorded to range from 16.5 to 217.3 mm, with an average annual rainfall of 116.9 mm^50^. Figure 2 provides the location of 222 historical landsides that were identified during comprehensive field survey and from areal imagery. Geologically, the study area is located on a plain with rocky outcrops and mountains towards the north-western and south-western parts. The Zagros suture zone is the result of a collision between the Arabian and Central Iran tectonic plates^51^. The main tectonic trend in the region follows the Zagros Mountains. The trend is aligned NW–SE and has affected the geo-structures in the region, including fault orientations, folding and shear zone formation^52^. Although most of the study area is covered by Quaternary sediments, the geological formations in the region include late Triassic rocks^53^. The geo-structures in the region can lead to different sliding and land-movement activities. The most important reasons for landslides in this province relate to tectonic structures rather than geological unit characteristics. Naturally, sedimentary rocks, especially marl formations, are more affected by landslides than igneous formations in the region, with the most important driving factors for landslides being tectonics and seismotectonics^54^.

#### Landslide covariates

The selection of a set of influencing factors is considered a key step in landslide susceptibility analysis^56^. Both full-length field surveys and remote sensing observations were acquired to provide a detailed landslide assessment of the study area. During the field surveys, 222 historic landslides in the study area were identified to determine landslide-prone areas. Several triggering factors, as used in numerous studies on machine learning-based landslide susceptibility modelling, categorized into several groups^57,58^, were used as landslide conditioning factors. The selection of the triggering factors required several considerations related to the dependency of triggering elements, measurability, non-redundancy and relevance of geological characteristics. The main factors influencing landslide occurrence were identified by preparing a spatial landslide inventory database that included the spatiotemporal distribution of historical landslides and a set of potential influencing factors. As a result, four main groups of factors were identified as the most effective elements that triggered landslide movements, including geomorphologic (i.e., altitude variation, slope aspect, slope curvature, profile curvature), geological (i.e., geo-units, distance to faults, land-use, soil type, hydrologic variation, slope-dip), environmental (i.e., climate, watershed, drainage pattern, vegetation) and human activity-related (i.e., distance to roads, distance to cities) covariates. These covariates were identified based on expert knowledge from fieldwork and remote sensing imagery. Table 1 provides information about the selected covariates used in this study. Before these data can be used in susceptibility modelling, it could be subject to multicollinearity and correlated variables^21^. The multicollinearity is a phenomenon in which one predictor variable in a regression model can be predicted linearly from others. To test for multicollinearity variance inflation factors (VIF) are commonly used^21,59^. A VIF > 5 indicates potential multicollinearity. In this article, all selected triggering factors produced VIF values less than 2.1 (Table 1).

**Table 1:** The information about landslide triggering factors used in this study.

#	Class	Triggering factors	Resolution	Variables	Data source	VIF
1	Geomorphologic	Altitude variation	± 30 m	Continuous	DEM	1.17
2		Slope aspect	± 30 m	Continuous	DEM	1.09
3		Slope curvature	± 30 m	Continuous	DEM	1.95
4		Profile curvature	± 30 m	Continuous	DEM	2.03
5	Geological	Geo-units	± 30 m	Continuous	Geological data	1.01
		Distance to faults	± 30 m	Continuous	DEM, Google Map	1.63
6		Land-use	± 30 m	Discrete	Landsat TM, ETM^+^	1.12
7		Soil type	± 30 m	Discrete	Landsat TM, ETM^+^	1.24
8		Hydrologic variation	± 30 m	Continuous	Landsat TM, IWRM*	1.86
9		Slope-dip	± 30 m	Continuous	DEM	1.13
10	Environmental	Climate	± 30 m	Discrete	Landsat ETM^+^, IMO^†^	1.09
11		Watershed	± 30 m	Discrete	Landsat TM, ETM^+^	1.45
12		Drainage pattern	± 30 m	Discrete	Landsat TM, ETM^+^	1.86
13		Vegetation (NDVI)	± 30 m	Continuous	Landsat TM, ETM^+^	1.20
14	Human activity	Distance to roads	± 30 m	Continuous	DEM, Google Map	1.17
15		Distance to cities	± 30 m	Continuous	DEM, Google Map	1.09

*Iran Water Resources Management Company (IWRM)^60^.

^†^Iran Meteorological Organization (IMO)^50^.

#### Data preparation

**Figure 3:**
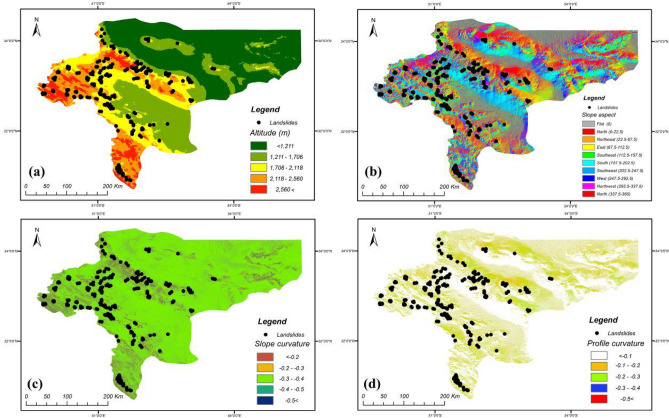
The geomorphologic factors used in the analysis: (**a**) altitude variation, (**b**) slope aspect, (**c**) slope curvature, (**d**) profile curvature using ArcGIS 10.4.1 software package^55^.

**Figure 4:**
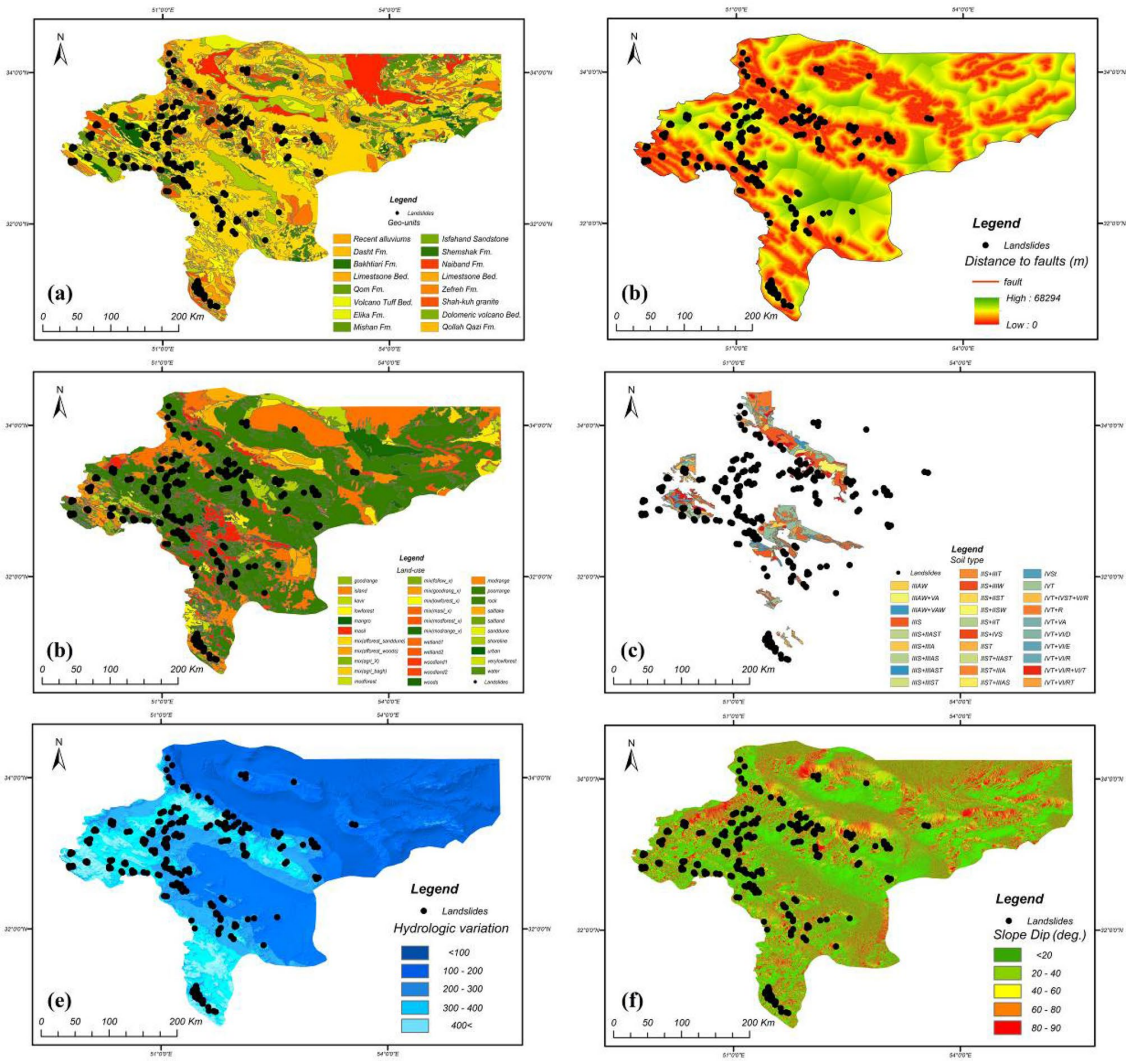
The geologic factors used in the analysis: (**a**) geo-units, (**b**) distance to faults, (**c**) land-use, (**d**) soil type, (**e**) hydrologic variation, (**f**) slope dip using ArcGIS 10.4.1 software package^55^.

**Figure 5:**
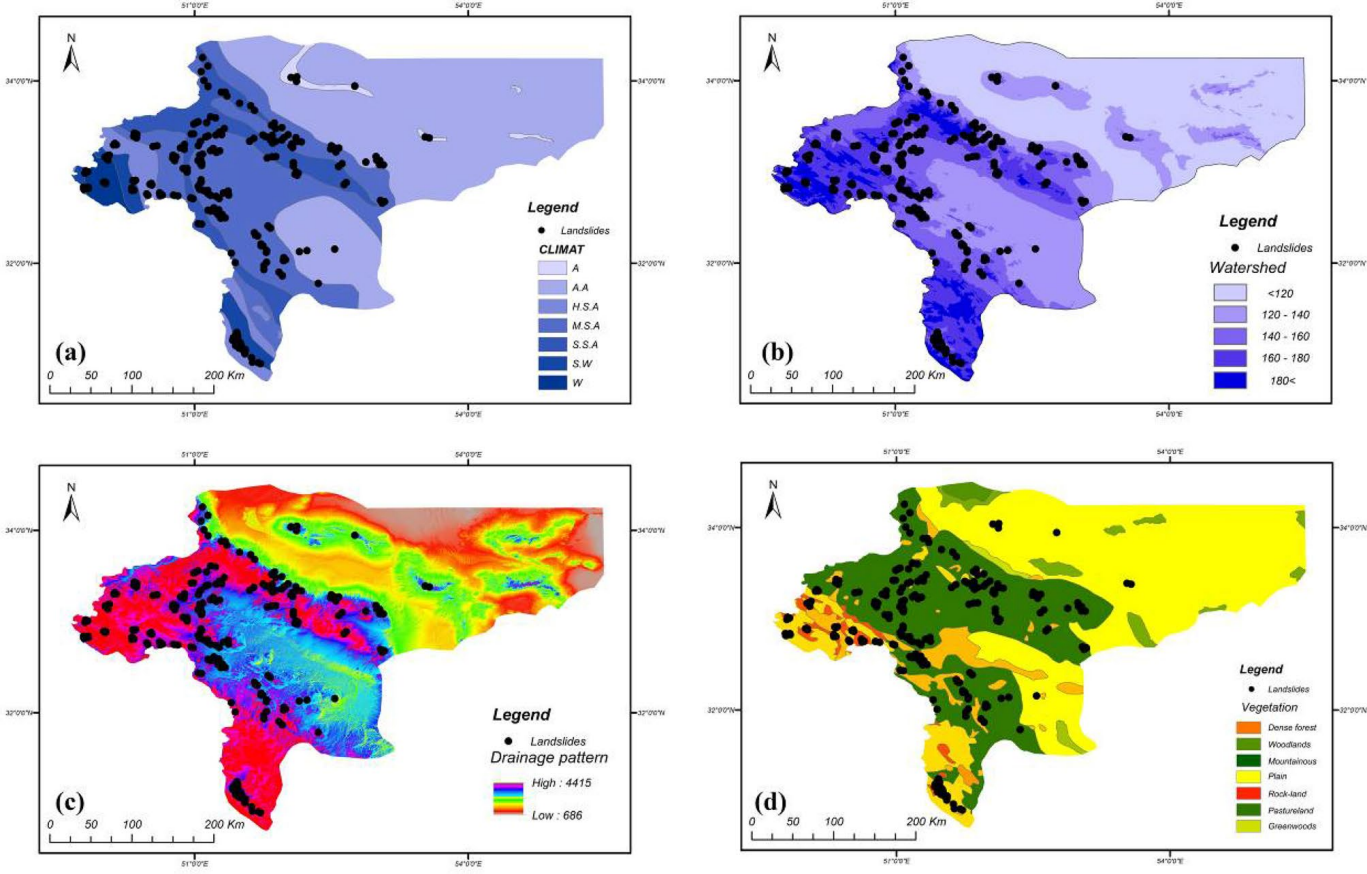
The environmental factors used in the analysis: (**a**) climate, (**b**) watershed, (**c**) drainage pattern, (**d**) vegetation using ArcGIS 10.4.1 software package^55^.

**Figure 6:**
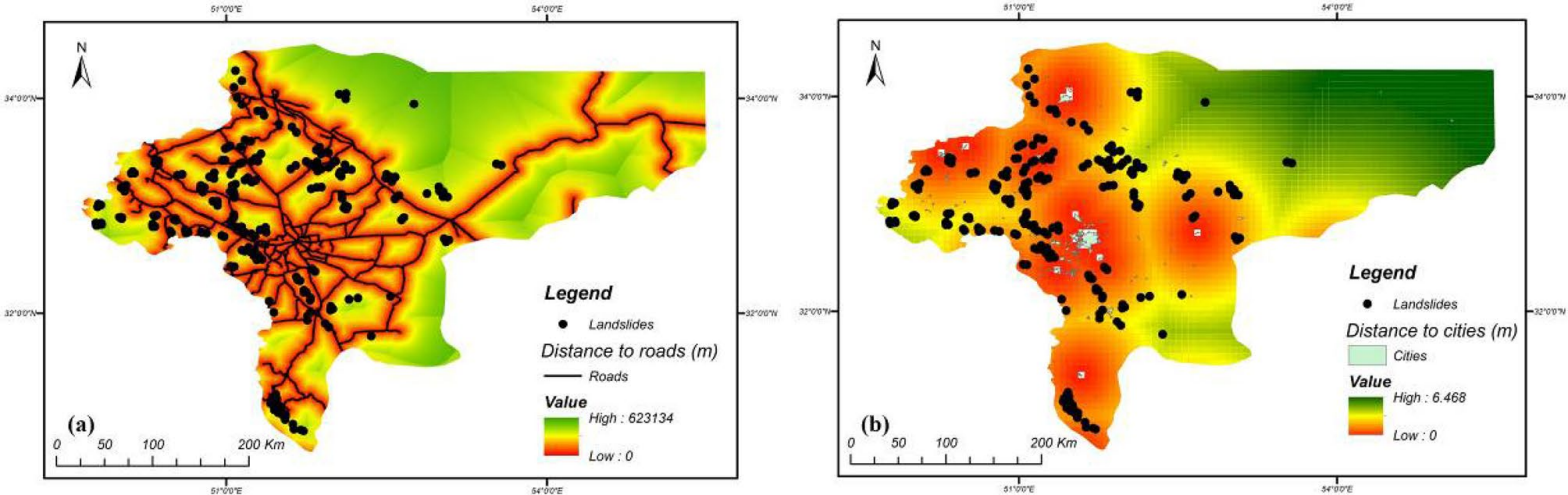
The human-activity related factors used in the analysis: (**a**) distance to roads, (**b**) distance to cities using ArcGIS 10.4.1 software package^55^.

In this research, four groups of covariates were considered for landslide susceptibility analysis. The inventory-based dataset was prepared using a digital elevation model (DEM) and Landsat TM (5–8), and ETM^+^ satellite sensor imagery provided by the Geotechnology Unit, Department of Geological Engineering, Middle East Technical University. The dataset included 222 recorded historical landside locations that were retrieved from technical documents, fieldwork and areal images taken from landsliding sites, checked using GPS coordinates and site-survey. The predictive models were fitted based on both landslide and non-landslide cells (i.e., where landslides did not occur). The flat plain area in Isfahan province was considered as contributing non-landslide cells (112 points in the dataset) mostly located in the east of the province. According to Huang et al.^33^, three methods exist for attaining non-landslide grid cells: the seed-cell procedure, random selection and flat locations (slope lower than 2°). This study used random selection, while including flat locations as well (due to the geomorphological condition of the province). After providing the main database, this database was divided into training and testing sets (80% and 20% of the information from the ground survey, respectively). The training set comprises 60% landslide − 40% non-landslide; while testing set comprises 55% landslide − 45% non-landslide. Figures 3, 4, 5 and 6 present maps of the landslide covariates to support visual assessment of the performance of the various methods tested. The ArcGIS v10.4 software was used to produce the landslide susceptibility maps. All evaluated spatial data were converted to spatially defined layers to produce the landslide susceptibility maps. The proposed algorithm was implemented in the Python high-level programming language. The results of the CNN–DNN evaluation were extracted as shapefiles and used as information layers in a GIS environment.

### Methodology

The study was conducted in several stages. First, ground survey was performed to estimate and record historical landslides in the study area. Second, by considering both the feature extraction of the CNN and the classification capabilities of the DNN, it was possible to identify highly susceptible (high risk) areas, potentially with high accuracy. In the next stage the model tested by using performance criteria, error models and the ROC curve.

This research evaluated the suitability of the proposed CNN–DNN method to produce detailed landslide susceptibility maps for the Isfahan province in Iran. The performance of the CNN–DNN was evaluated against several high-quality benchmark approaches through a range of appropriate statistical measures. A total of 15 landslide covariates, falling into four main groups, were fed into the CNN–DNN. All covariate layers were normalised and then entered into the model to standardise and prepare the information for landslide susceptibility analysis. The CNN was used for feature extraction, and the DNN was used to sort pixels into the high-susceptible and low-susceptible groups. Table 2 provides the hyperparameters used in the study. Hyperparameters are commonly used to optimize the fitting process which can increase the machine learning model prediction accuracy^21^. The objective of selecting the hyperparameters is to optimize the evaluation values^38,61^. Different optimisers were used for the hyperparameters, noting that some optimizers provide more accurate results than others^61^. The presented study used the grid search technique for the assessments. The hyperparameters that provide the highest accuracy were chosen for the final training and testing of the respective machine learning models^21^.

**Table 2:** Hyperparameters for optimal values of the machine learning-based models.

Classifier	Hyperparameters	Explanation
SVM	Kernels	Gama, Linear
	C value	Regularization intensity value
LR	Random state	Controls the randomness
	Max iter	Maximum number of iterations
GNB	Smoothing	Largest variance of all features that is added to variances for calculation stability
MLP	Hidden layers’ size	Hidden layers’ number
	Learning rate	Adaptiveity of learning rate
BNB	Alpha	Additive smoothing parameter
DT	Max depth	Maximum depth of the tree
	Random state	Controls the randomness of the estimator
CNN–DNN	Learning rate	Adaptiveity of learning rate
	Number of hidden layers	Hidden layers’ number
	Units	Unit for each layer
	Batch size	The number of training examples used in the estimate of the error gradient

**Figure 7:**
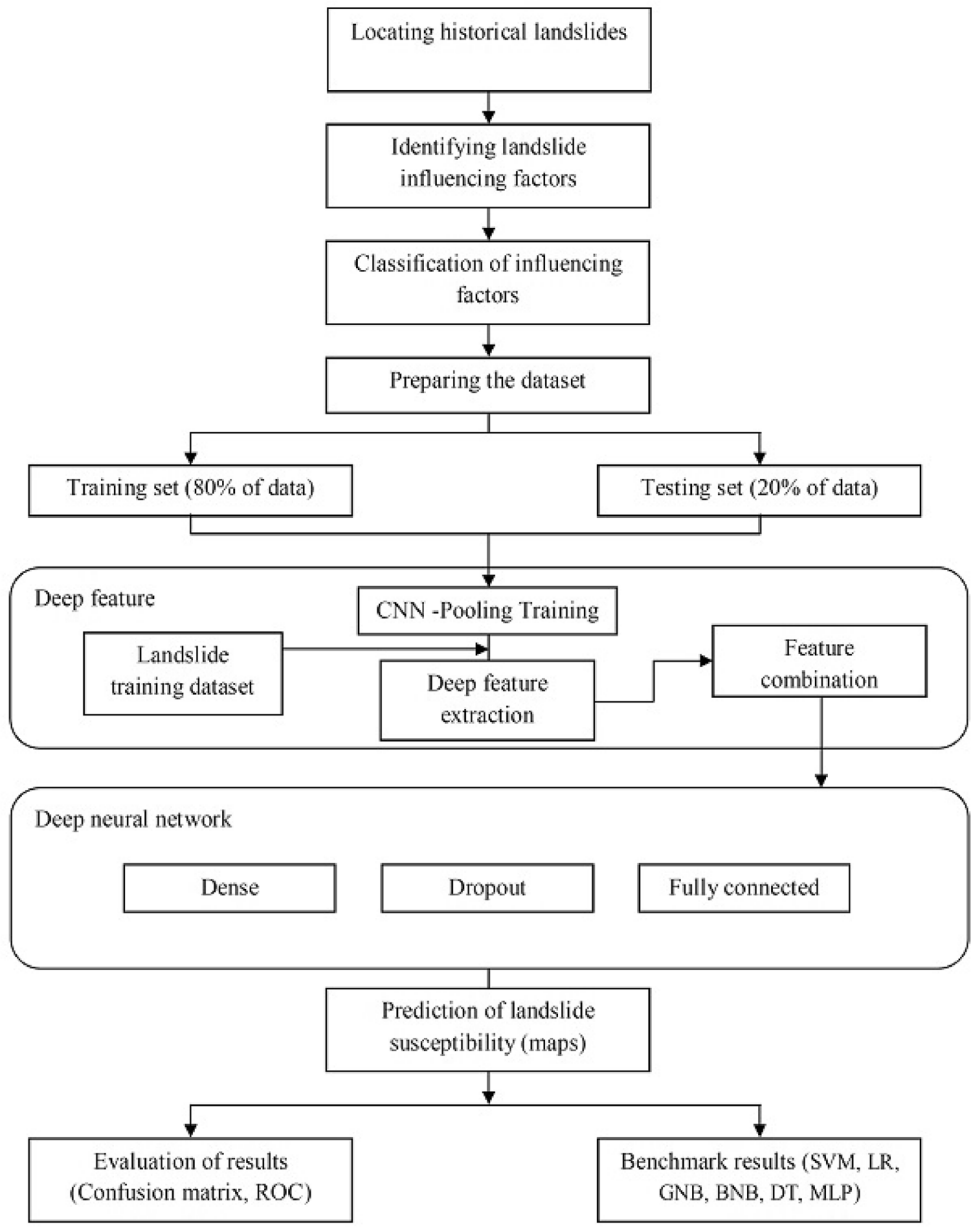
The processing flowchart of the proposed model.

Figure 7 presents a flowchart describing the process applied for susceptibility assessment. As seen in the figure, the landslides dataset includes 222 historical cases and field survey recordings divided randomly into training (80%) and testing (20%) datasets. The database consists of the landslide inventory datasets (training and validation) and the landslide triggering factors. These factors were subsequently evaluated by calculating their weights from the relationship between the landslide occurrences and landslide triggering factors and then these results were checked^62^. There is no standard for the selection of triggering factors in susceptibility mapping, but the chosen factors have to be measurable depending on a particular area’s characteristics^63^. As mentioned, the test and train datasets represented 20–80%, respectively, of the primary database, taking their spatial distributions into account. Considering the test/train ratio is important for the model learning rate, that is, the response to the estimated error each time the model weights are updated. In fact, the learning rate controls how quickly the model is adapted to the problem. Smaller learning rates require more training epochs as smaller changes are made to the weights at each update, whereas larger learning rates result in rapid changes and require fewer training epochs. Specifically, the learning rate is a configurable hyperparameter used in the training of neural networks that has a small positive value, often in the range between 0.0 and 1.0. The learning rate used in this study was selected by optimizers, which for 0.01 and no momentum were scheduled via callbacks in Keras support. Pearson's Phi coefficient was used to assess the susceptibility classification, and each of the landslide influencing factors was used in this process. The coefficient takes into account true and false positives and negatives. It is generally a balanced measure that can be used even if the class proportions are of very different sizes.

**Figure 8:**
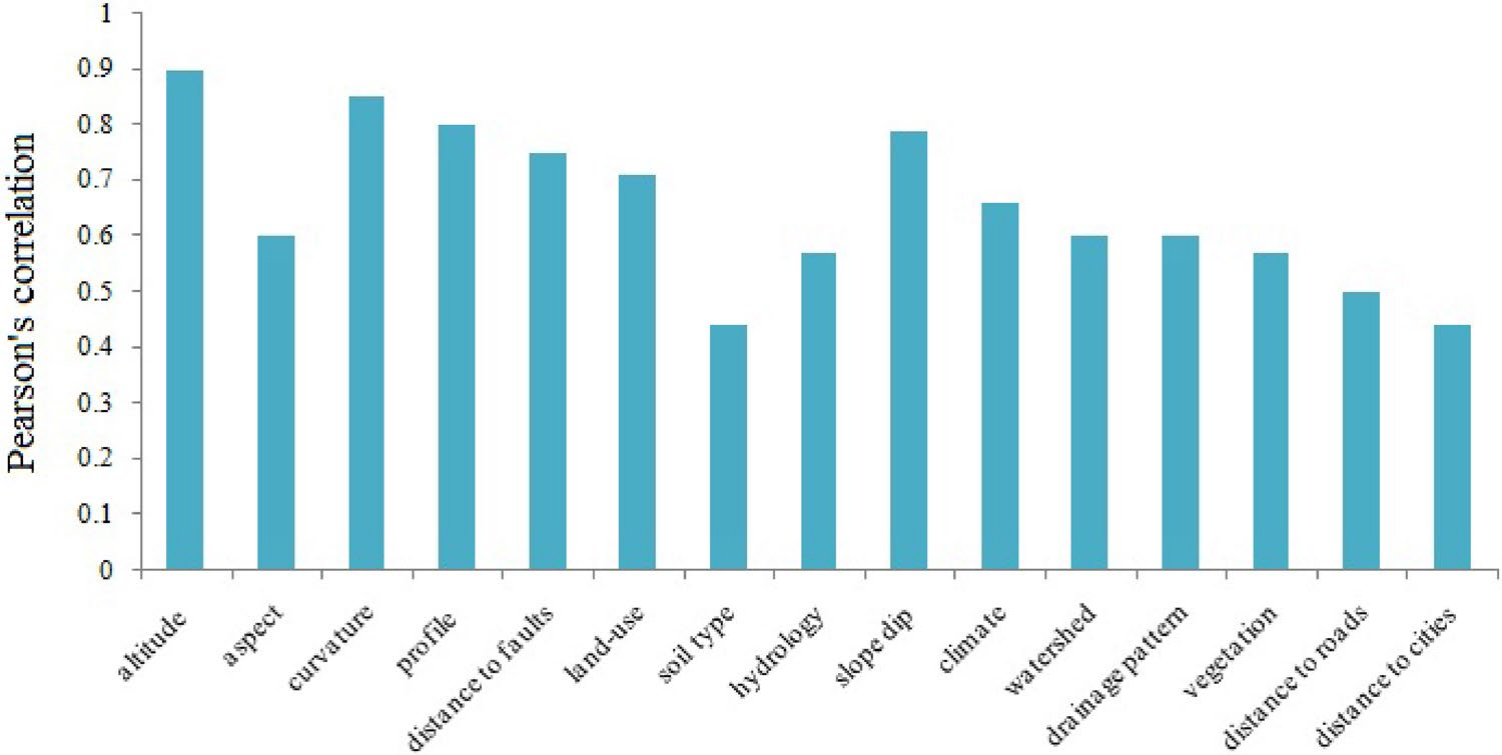
Pearson's coefficient for each information layer.

Figure 8 presents the Pearson's coefficients for each layer. These information layers constituted the landslide dataset and were input to the CNN to extract more informative features for susceptibility assessment. These feature representations were then used in the DNN model to produce the susceptibility map. As is well known, some of the covariates, such as land cover, can be accepted by the proposed method, but some others, such as land use, must be modified before input to the CNN–DNN. In this regard, we used the class weight argument in the Keras package to select a large weight for unbalanced classes in such factors (to produce balanced values).

This classification is based on a set of influencing factors (which cover extrinsic and intrinsic elements) trained on historical landslide occurrences (a total of 222 landslides) characterising very high and high susceptibility zones. The historical landslide data were prepared and extracted from shapefiles implemented in a GIS environment and evaluated for each input factor.

To assess the proposed methodology rigorously, its accuracy was evaluated using statistics from the OA and ROC and compared with the accuracies of common machine learning methods, including the SVM, LR, GNB, MLP, BNB and DT classifiers. From the confusion matrix, the mean squared error (MSE), root mean square error (RMSE) and mean absolute percentage error (MAPE) were used to measure the model accuracy. In this regard, the algorithm was run for 5000 iterations (epochs) using the training and validation datasets. The stochastic gradient descent (SGD), RMSprop and Adagrad high-dimensional optimisers were used as objective functions with suitable smoothness properties to provide accurate results. This helped to reduce the computational burden by balancing the number of iterations against the convergence rate.

The performance of the proposed methodology was estimated based on both the confusion matrix and the algorithm performance matrix. The performance matrix is a specific table that visualises the performance of a prediction algorithm based on its predicted values, and it contains the sensitivity, specificity and 1-specificity parameters. For classification tasks, true positives (TP), true negatives (TN), false positives (FP) and false negatives (FN) are used to compare the results of the classifier in question with trusted external judgments (Hearty 2016). Precision, also called the positive predictive value, is the fraction of relevant instances (TP) amongst the retrieved instances.

1 $${\text{Precision = }}\frac{{{\text{TP}}}}{{{\text{TP }} + {\text{FP}}}}\,$$

Recall (sensitivity) is the total fraction of relevant instances.

2 $${\text{Recall/Sensitivity = }}\frac{{{\text{TP}}}}{{{\text{TP}} + {\text{FN }}}}$$

Therefore, both precision and recall are based on measures of relevance^41^. The false-positive rate can be calculated as ‘1-specificity’, where specificity is defined as:

3 $${\text{Specificity = }}\frac{{{\text{TN}}}}{{{\text{TN}} + {\text{FP }}}}$$

Accuracy can be a misleading metric for imbalanced datasets. For example, for a prediction set with 95 positive and 5 negative values, classifying all values as negative gives a 0.95 accuracy score. On the other hand, the F1-score, the harmonic mean of precision and recall, provides approximately the average of the two values when they are close and is more generally the harmonic mean.

4 $${\text{F1 - score = 2}} \cdot \frac{{{\text{Precision}} \cdot {\text{ Recall}}}}{{\text{Precision + Recall}}}$$

The overall accuracy (OA) represents the probability that a test will correctly classify an individual; that is, the sum of TP plus TN divided by the total number of the individuals tested:

5 $${\text{Accuracy = }}\frac{{{\text{TP}} + {\text{TN}}}}{{{\text{TP}} + {\text{TN}} + {\text{FP}} + {\text{FN}}}}$$

OA is, thus, also the weighted average of ‘sensitivity’ and ‘specificity’ (Aggarwal, 2018). The application of the performance matrix helps to characterise the trustworthiness of the classifier in question.

## Results

### The proposed models

**Figure 9:**
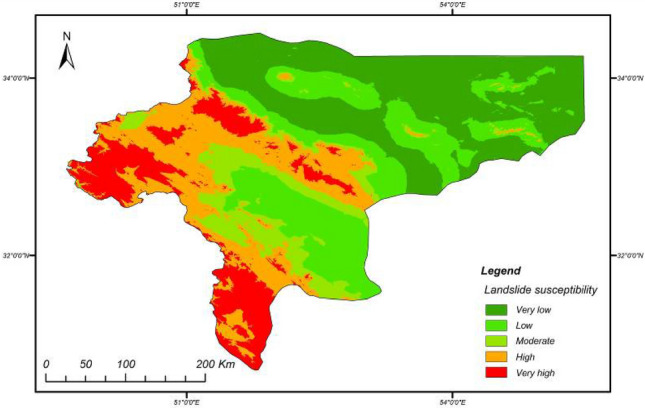
Landslide susceptibility map for the proposed model using ArcGIS 10.4.1 software package^55^.

**Figure 10:**
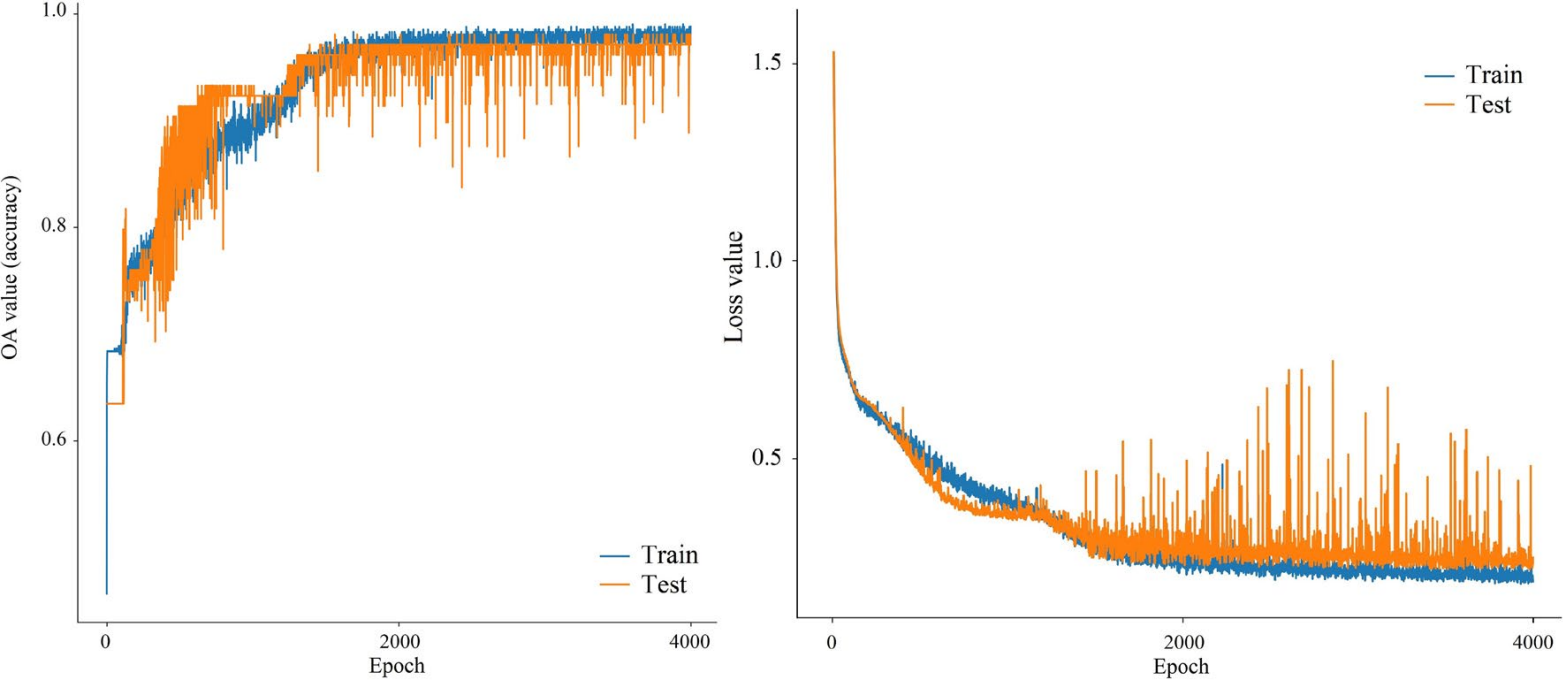
The OA and loss function values were obtained for the applied model.

**Figure 11:**
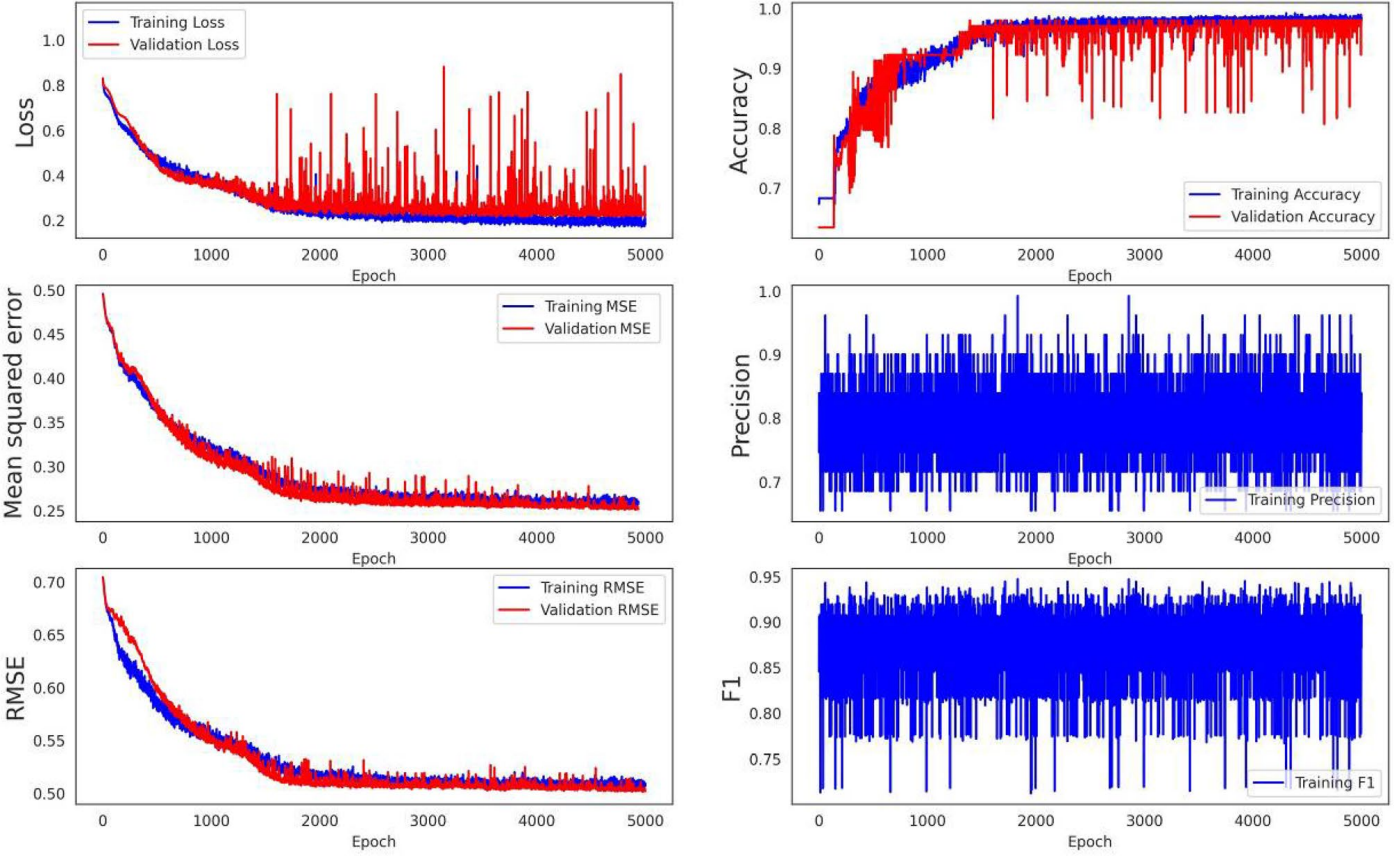
The SGD optimiser results for the proposed model.

**Figure 12:**
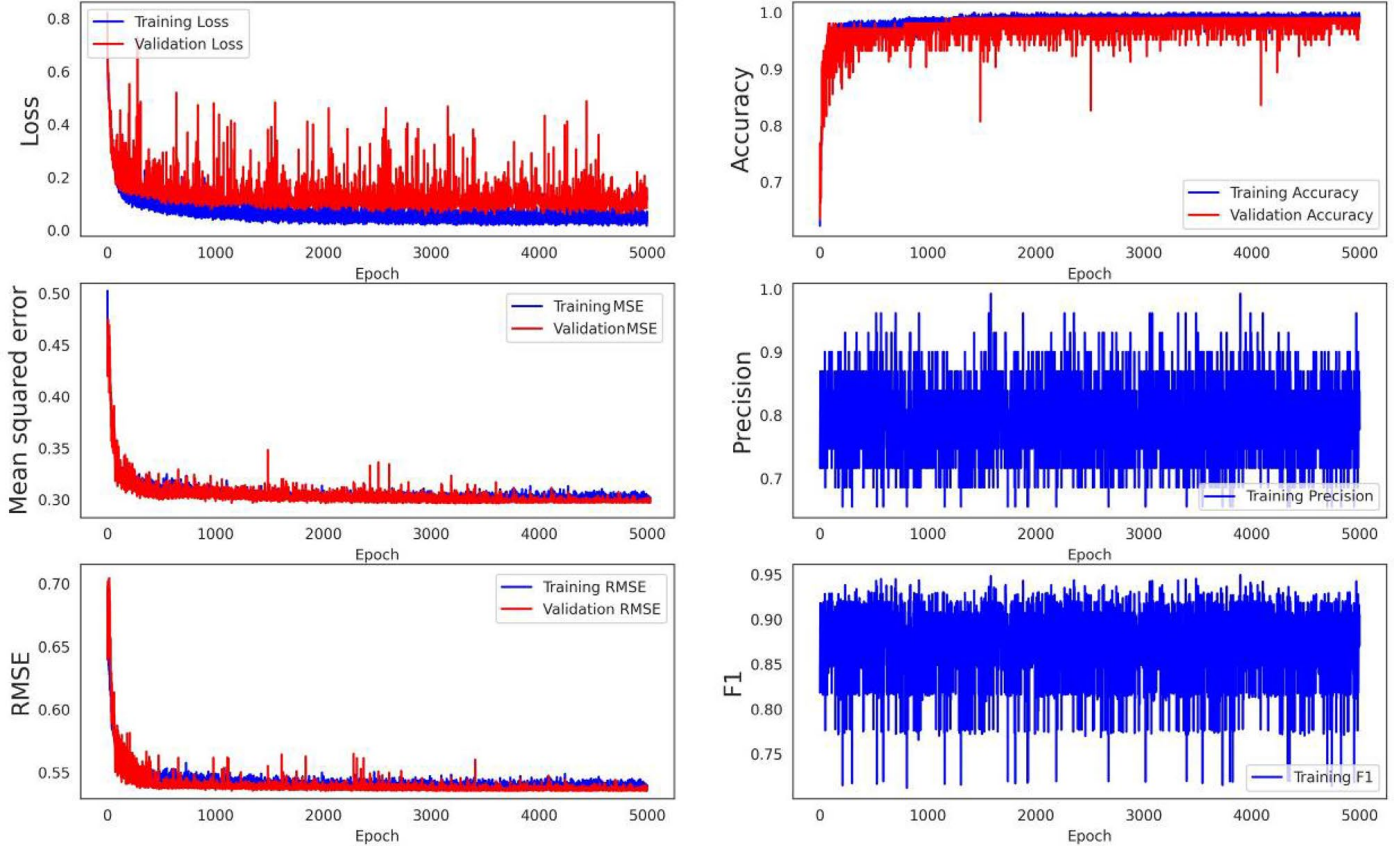
The RMSprop optimiser results for the proposed model.

**Figure 13:**
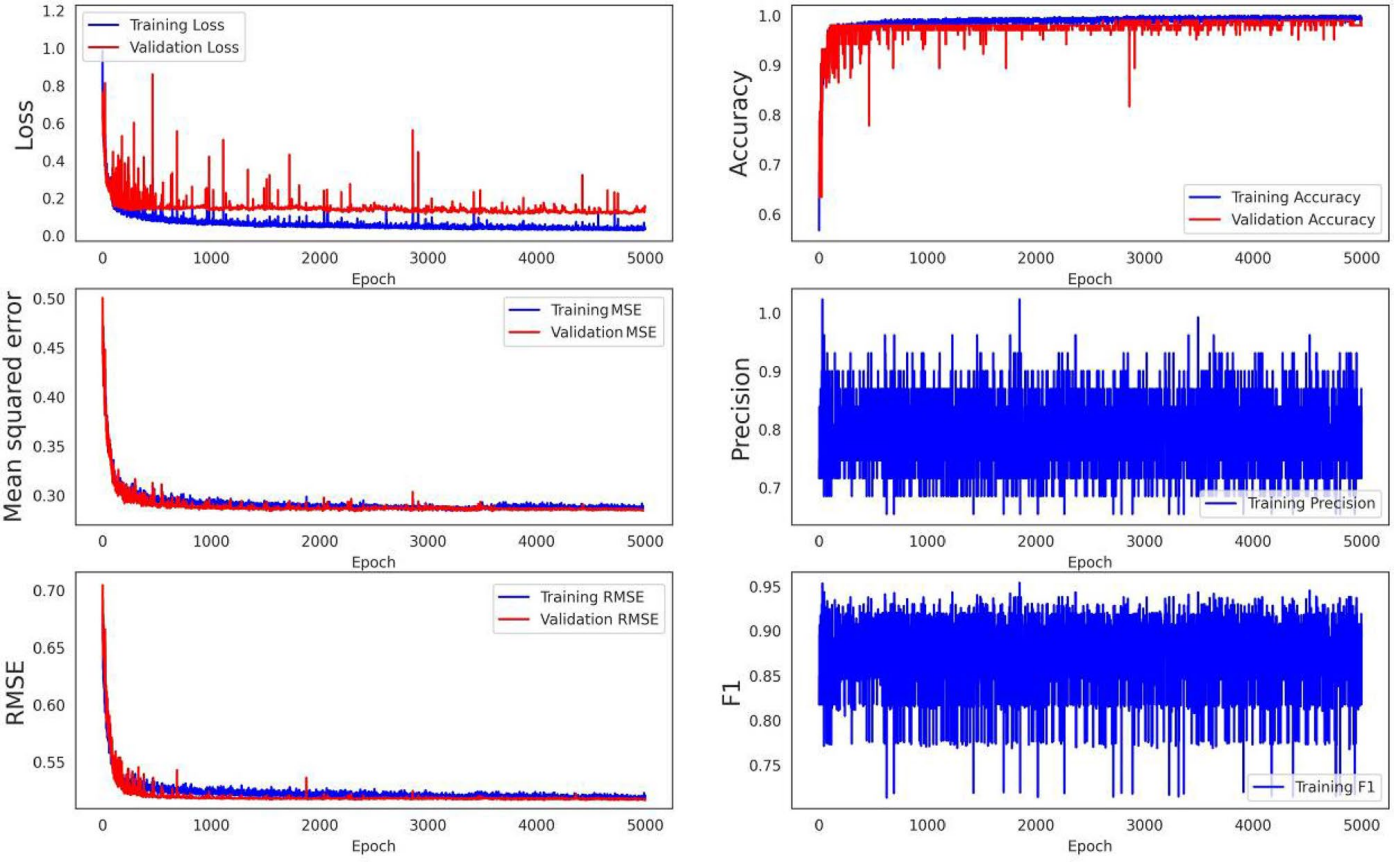
The Adagrad optimiser results for the proposed model.

Landslide hazard susceptibility assessment was conducted by applying the proposed CNN–DNN methodology to evaluate landslide susceptibility in the study area (Fig. 9). The OA and ROC controlled the result of the proposed model. The ROC curve is a graphical description that shows the diagnostic ability of a binary classifier system as its discrimination threshold is varied. As a result, the OA and AUC from the ROC curve represent the accuracy of the classifiers. Figure 10 presents the OAs and loss models for the CNN–DNN. These figures show that the estimated OA is 0.909, and the loss is reduced to 0.20 in 5000 epochs. According to the evaluated hazard map presented in Fig. 9, susceptible and hazardous areas in the west and southern parts of the Isfahan province manifest spatially as visual stripes. DNN optimisers such as SGF, RMSprop and Adagrad estimated the modified IRs for the CNN–DNN (Figs. 11, 12, 13). IRs can be used as the AUC value of OA to control the performance of the algorithm. These stripes follow the northwest-southeast trace, which represents the main Zagros trend in the region. Therefore, it can be suggested that geological factors have had the most significant impact on landslide occurrence in the Isfahan province.

### Benchmark comparison

**Figure 14:**
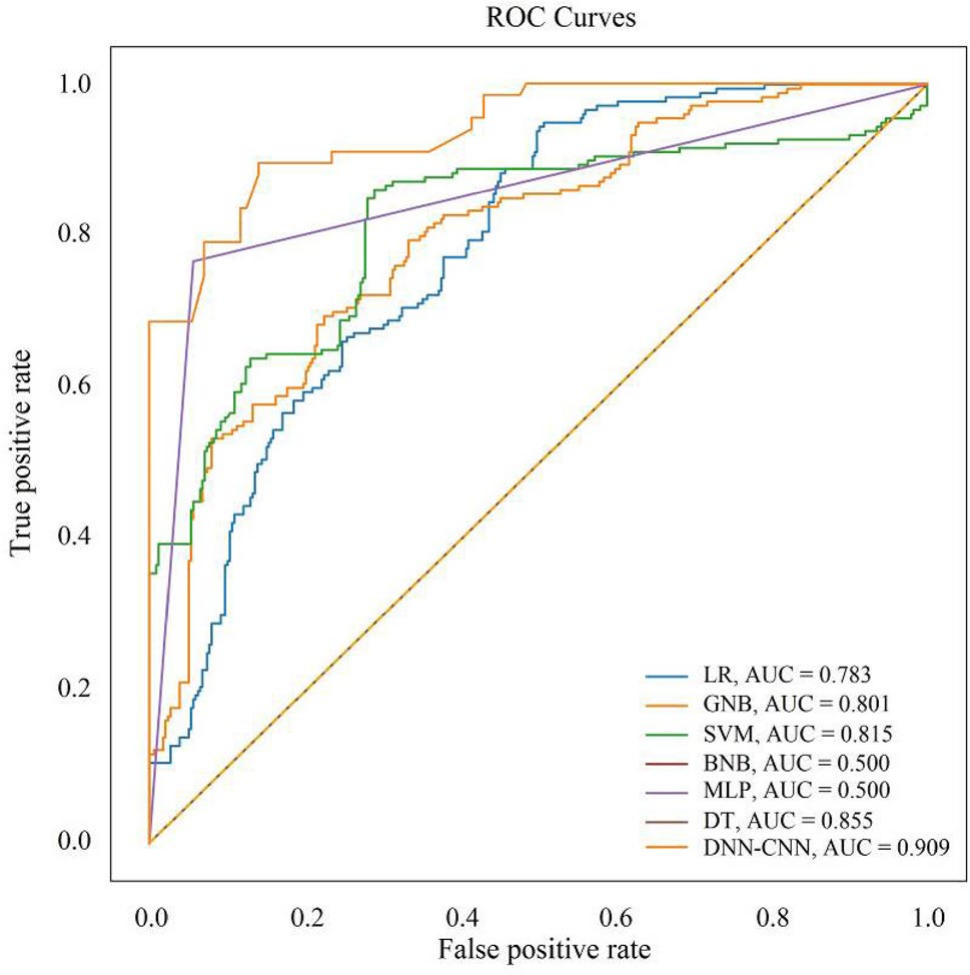
ROC results for the CNN–DNN model and the benchmark methods.

To evaluate the performance of the proposed CNN–DNN model rigorously, a large range of state-of-the-art and widely applied machine learning techniques were tested using the same accuracy statistics as applied to the proposed method. These benchmark methods include the SVM, LR, GNB, MLP, BNB and DT classifiers. Thus, the OA, IRs and ROC were also obtained for these benchmark methods (Fig. 14 and Table 3). By comparing the accuracy evaluation for the CNN–DNN with those for the benchmark approaches, it can be observed that the CNN–DNN model was able to predict landslide susceptibility with higher accuracy than the other classifiers. The IRs and AUC estimated for the CNN–DNN and benchmark methods indicate that the CNN–DNN method has significantly greater accuracy than the benchmarks. This suggests that the extracted features can more accurately characterise landslide susceptibility than the benchmark methods as measured with the AUC and IR indices. More specifically, the CNN–DNN (AUC = 90.9%; IRs = 84.8%) achieved greater prediction accuracy than the corresponding single classifiers such as SVM (AUC = 81.5%; IRs = 80.1%), LR (AUC = 78.3%; IRs = 72.2%), GNB (AUC = 80.1%; IRs = 68.7%), BNB (AUC = 50.0%; IRs = 61.0%), MLP (AUC = 50.9%; IRs = 61.8%) and DT (AUC = 85.5%; IRs = 80.0%) as revealed through the measured indices. The MSE, RMSE and MAPE values were also obtained for the various classifiers (Table 4). According to this table, the CNN–DNN model outperformed the benchmark methods.

**Table 3:** The performance matrix for further predictive analysis.

Classifier	Parameter	Assessment score				Accuracy	AUC (ROC)
		Precision	Recall	F1-score	Support		
SVM	High-susceptible	0.81	0.80	0.80	98	0.801	0.815
	Low-susceptible	0.81	0.82	0.81	98		
LR	High-susceptible	0.74	0.71	0.72	98	0.722	0.783
	Low-susceptible	0.74	0.74	0.74	98		
GNB	High-susceptible	0.69	0.67	0.68	98	0.687	0.801
	Low-susceptible	0.70	0.70	0.70	98		
MLP	High-susceptible	0.31	0.50	0.38	98	0.610	0.500
	Low-susceptible	0.37	0.61	0.46	98		
BNB	High-susceptible	0.31	0.50	0.38	98	0.618	0.500
	Low-susceptible	0.37	0.60	0.46	98		
DT	High-susceptible	0.80	0.82	0.80	98	0.800	0.855
	Low-susceptible	0.79	0.81	0.80	98		
CNN–DNN	High-susceptible	0.83	0.92	0.87	130	0.848	0.909
	Low-susceptible	0.86	0.84	0.84	130

**Table 4:** The model error is estimated for different predictive accuracy functions.

Classifier	MSE	RMSE	MAPE
SVM	0.28367346	0.42857142	0.44225426
LR	0.25510204	0.50507627	0.54267105
GNB	0.29591836	0.54398379	0.63293793
MLP	0.38775510	0.62269984	0.65037790
BNB	0.38775510	0.62490772	0.65269613
DT	0.28073840	0.42631849	0.44543989
CNN–DNN	0.17112569	0.40582108	0.42187734

As Table 3 shows the values of the classification metrics, the proposed model performed more accurately than all six benchmarks in all metrics. The proposed model produced the highest rate of ROC accuracy with a value of 90%. After the proposed model, the decision tree classifier achieved the next best performance, with accuracy approximately 5% lower than for the proposed model. The lowest estimated accuracy of 50% was achieved by the MLP and BNB, which is 40% less than for the proposed model. Regarding accuracy criteria, the proposed model produced an accuracy of 84.8%, and the closest algorithm (SVM) to the proposed model was approximately 4.7 less than the proposed model. The MLP produced the lowest accuracy of 61%. The average precision for the two susceptibility classes of the proposed model is 84%. The lowest average accuracy of the MLP and BNB is 34% (a difference from the proposed model of more than 50%). The average recall rate for the proposed model is 88%, and the minimum recall rate is 55% for BNB. For the F1-score, the average is 85.5% for the proposed model, and, as for the other three criteria examined, this is the largest value amongst all models. The DT algorithm produced the next largest F1-score, with an average value of 83.5% and a difference of almost 2% less than the proposed model.

## Discussion

We investigated the potential of a coupled deep neural network (CNN–DNN) to predict landslide susceptibility spatially. The algorithm was evaluated using data with a spatial resolution of 30 m representing the Isfahan province, Iran. Indices associated with historical landslide occurrences (a total of 222 landslides) were used as the landslide inventory dataset, and this was divided randomly into training (80%) and testing (20%) sets for the analysis. Four main covariates, including geomorphologic, geologic, environmental and human activity-related covariates, were identified based on field and remote sensing investigations. The CNN–DNN model was able to produce a susceptibility map for the study area with appropriate accuracy. The results show a significant increase in landslide susceptibility prediction accuracy compared to the benchmark models. Notwithstanding the high accuracy achieved by the proposed CNN–DNN predictive model for landslide susceptibility mapping, this study has some limitations that could be considered in future research. Theses limitation can be addressed as: (*i*) the primary database was provided based on fieldwork, historical landslide records and remote-sensing information. The limited number of reference landslides in the recorded data (as is commonly the case) made modelling challenging; (*ii*) the data on the triggering factors were highly dependent on the spatial resolutions of satellite sensor imagery and DEM data quality, which affected directly the quality of the input database; (*iii*) the predictive model required strong processors to manage the inputs during landslide susceptibility assessments. Thus, for future scientific research involving, for example, even finer spatial resolution images the adequacy of the available processors needs to be considered for landslide susceptibility analysis.

Referring to Fig. 9, which presents the landslide susceptibility assessment results in the study area, it is clear that the main risk area lies in the west and southwest part of Isfahan. Geo-structural studies suggest that the high-susceptible areas are located in the Zagros folded zone and follow the main Zagros trend in the province. Thus, it can be stated that the geological-based triggering factors play important roles in determining landslide occurrence in Isfahan. Fieldwork suggested the effect of geo-structures as triggers of landslide movements. It is interesting then that the CNN–DNN model was able to provide detailed mapping to corroborate this.

The benchmark classifiers SVM, LR, GNB, MLP, BNB and DT were used to validate the predictive performance of the CNN–DNN model. Comparison of the proposed model with the benchmark methods demonstrated the superiority of the proposed CNN–DNN approach. A review of recent studies on landslide susceptibility assessment demonstrated that applications of deep neural networks in susceptibility analysis are expanding^37,42,44,45^. Wang et al.^42^, Sameen et al.^37^, Fang et al.^32^, and Pham et al.^8^ used a CNN as the principle method for the assessment of landslide susceptibility for different locations, with an evaluation accuracy of 0.813, 0.835, 0.798, and 0.889; respectively^8,32,42,44^. This indicates that the CNN can be used as a basic predictive model. However, the coupled CNN–DNN model in this paper was able to increase the accuracy further, to reach 0.909.

The CNN–DNN method uses a first-stage CNN component that attempts to extract meaningful semantic information from low-level input covariates that may be related to the target for prediction, in this case, landslide susceptibility classes. The results suggest that the first-stage CNN is efficient in extracting suitable environmental features related to landslide susceptibility. This is important because it is unclear whether landslides should be considered as spatially continuous phenomena or spatial objects^18–20,26–29,64^. On the one hand, landslides are complex geomorphological processes manifested as changes in states in space and time, including variation within the landslide (rupture zone and impacted area). Thus, at a fine spatial scale, one might consider a continuous statistical model appropriate for landslide susceptibility mapping. On the other hand, landslides create discrete rupture zones and impacted areas that appear against a landscape background. In this sense, and at a scale where variation between landslides becomes more important, landslides can be considered discrete objects.

The problem with the above duality between the continua- and object-based views of the world becomes obvious when considering the characterisation of existing landslides and prediction of yet-to-occur landslides. Landslides do not occur at a pixel, but rather occupy some positive area. As such, conventional methods, which are commonly pixel-based, insufficiently characterise the landslide as a spatially extensive phenomenon. They also run into difficulties in predicting yet-to-occur landslides because predictions of susceptibility are constrained to a pixel. The CNN–DNN model deals directly with these two problems by analysing spatial patches of data rather than pixels.

The second problem we leave as an open question for future research. Specifically, the CNN–DNN can transform the spatial information in the input covariates into meaningful higher-order feature representations about landslide susceptibility. This makes sense concerning landslides when one considers the conditions that may lead to failure. These conditions are often spatial, requiring not the conditions at a point to be satisfied, but the conjunction of several conditions over an area to be satisfied. For example, it may not be enough for the slope at a single point to be high. Landsliding may be more likely if that same high slope falls in the context of surrounding land which, for example, concentrates water to that point (e.g., via overland flow or throughflow). This requirement for context is true of many of the in situ factors that underpin the susceptibility of a location to fail. Thus, the CNN–DNN approach proposed in this research is an excellently matched algorithm to the specific characteristics of the landsliding phenomenon and problem under study.

## Conclusions

Landslide susceptibility mapping is one of the most challenging tasks in geo-hazard assessment. In this context, application of modern deep learning techniques can be advantageous for analysis. Here, we applied a novel CNN–DNN predictive model for assessment of landslide susceptibility in Isfahan province, Iran. The model was fitted between historical landslides data (which accounted for different types of landsliding) and various triggering factors. The proposed CNN–DNN model produced a very high accuracy, outperforming a wide range of benchmark approaches, specifically the SVM, LR, GNB, MLP, BNB and DT methods. More specifically, the CNN–DNN (AUC = 90.9%; IRs = 84.8%) achieved greater prediction accuracy than the corresponding single classifiers such as SVM (AUC = 81.5%; IRs = 80.1%), LR (AUC = 78.3%; IRs = 72.2%), GNB (AUC = 80.1%; IRs = 68.7%), BNB (AUC = 50.0%; IRs = 61.0%), MLP (AUC = 50.9%; IRs = 61.8%) and DT (AUC = 85.5%; IRs = 80.0%) as revealed through the measured indices. Also, the CNN–DNN (MSE = 0.17, RMSE = 0.40, MAPE = 0.42) produced smaller error indices than the benchmark models: SVM (MSE = 0.28, RMSE = 0.42, MAPE = 0.44), LR (MSE = 0.25, RMSE = 0.50, MAPE = 0.54), GNB (MSE = 0.29, RMSE = 0.54, MAPE = 0.63), BNB (MSE = 0.38, RMSE = 0.62, MAPE = 0.65), MLP (MSE = 0.38, RMSE = 0.62, MAPE = 0.68), and DT (MSE = 0.28, RMSE = 0.42, MAPE = 0.44). We, thus, recommend the CNN–DNN approach for landslide susceptibility mapping. Importantly, the CNN component of the approach has great advantages for landslide susceptibility mapping precisely because it matches well, and takes advantage of, the spatially extensive nature of the landslide phenomenon itself.

The CNN–DNN model predicted a high-susceptibility zone in the west and south-western parts of the study area, appearing as a stripe aligned with the northwest-southeast main Zagros trend in the region.

## References

[1] Colesanti C, Wasowski J (2006). Investigating landslides with space-borne Synthetic Aperture Radar (SAR) interferometry. Eng. Geol..

[2] Highland L, Bobrowsky PT (2008). The Landslide Handbook: A Guide to Understanding Landslides.

[3] Chen Z (2016). Landslide research in China. Q. J. Eng. Geol. Hydrogeol..

[4] Tang H, Wasowski J, Juang CH (2019). Geohazards in the three Gorges Reservoir Area, China-Lessons learned from decades of research. Eng. Geol..

[5] Wasowski J (2021). Recurrent rock avalanches progressively dismantle a mountain ridge in Beichuan County, Sichuan, most recently in the 2008 Wenchuan earthquake. Geomorphology.

[6] Azarafza M, Ghazifard A, Akgün H, Asghari-Kaljahi E (2018). Landslide susceptibility assessment of South Pars Special Zone, southwest Iran. Environ. Earth Sci..

[7] Cascini L (2008). Applicability of landslide susceptibility and hazard zoning at different scales. Eng. Geol..

[8] Pham VD, Nguyen Q-H, Nguyen H-D, Pham V-M, Bui Q-T (2020). Convolutional neural network: Optimised moth flame algorithm for shallow landslide susceptible analysis. IEEE Access.

[9] Abella EAC, Van Westen CJ (2008). Qualitative landslide susceptibility assessment by multicriteria analysis: a case study from San Antonio del Sur, Guantánamo, Cuba. Geomorphology.

[10] Lee S, Choi J (2004). Landslide susceptibility mapping using GIS and the weight-of-evidence model. Int. J. Geogr. Inf. Sci..

[11] Manzo G, Tofani V, Segoni S, Battistini A, Catani F (2013). GIS techniques for regional-scale landslide susceptibility assessment: The Sicily (Italy) case study. Int. J. Geogr. Inf. Sci..

[12] Feizizadeh B, Blaschke T (2014). An uncertainty and sensitivity analysis approach for GIS-based multicriteria landslide susceptibility mapping. Int. J. Geogr. Inf. Sci..

[13] Firomsa M, Abay A (2019). Landslide assessment and susceptibility zonation in Ebantu district of Oromia region, western Ethiopia. Bull. Eng. Geol. Environ..

[14] Milevski I, Dragićević S (2019). Landslides susceptibility zonation of the territory of north macedonia using analytical hierarchy process approach. Contrib. Sect. Nat. Math. Biotechn. Sci..

[15] Peethambaran B, Anbalagan R, Kanungo D, Goswami A, Shihabudheen K (2020). A comparative evaluation of supervised machine learning algorithms for township level landslide susceptibility zonation in parts of Indian Himalayas. CATENA.

[16] Fang Z, Wang Y, Peng L, Hong H (2021). A comparative study of heterogeneous ensemble-learning techniques for landslide susceptibility mapping. Int. J. Geogr. Inf. Sci..

[17] Yan Y (2020). Volunteered geographic information research in the first decade: A narrative review of selected journal articles in GIScience. Int. J. Geogr. Inf. Sci..

[18] Rahman M, Chen N, Islam MM, Mahmud GI, Pourghasemi HR, Alam M, Rahim MA, Baig MA, Bhattacharjee A, Dewan A (2021). Development of flood hazard map and emergency relief operation system using hydrodynamic modeling and machine learning algorithm. J. Clean. Prod..

[19] Rahman M, Chen N, Islam MM, Dewan A, Iqbal J, Muhammad R, Washakh A, Shufeng T (2019). Flood susceptibility assessment in Bangladesh using machine learning and multi-criteria decision analysis. Earth Syst. Environ..

[20] Dewan A.M., Hazards, risk, and vulnerability. In: *Floods in a Megacity*, 35–74. 10.1007/978-94-007-5875-9_2 (2013).

[21] Adnan MSG, Rahman MS, Ahmed N, Ahmed B, Rabbi MF, Rahman RM (2020). Improving spatial agreement in machine learning-based landslide susceptibility mapping. Remote Sens..

[22] Zêzere J, Pereira S, Melo R, Oliveira S, Garcia RA (2017). Mapping landslide susceptibility using data-driven methods. Sci. Total Environ..

[23] Huabin W, Gangjun L, Weiya X, Gonghui W (2005). GIS-based landslide hazard assessment: an overview. Prog. Phys. Geogr..

[24] Ruff M, Czurda K (2008). Landslide susceptibility analysis with a heuristic approach in the Eastern Alps (Vorarlberg, Austria). Geomorphology.

[25] Nefeslioglu H, Sezer E, Gokceoglu C, Bozkir A, Duman T (2010). Assessment of landslide susceptibility by decision trees in the metropolitan area of Istanbul, Turkey. Math. Probl. Eng..

[26] Atkinson PM, Massari R (2011). Autologistic modelling of susceptibility to landsliding in the Central Apennines, Italy. Geomorphology.

[27] Eker AM, Dikmen M, Cambazoğlu S, Düzgün ŞH, Akgün H (2015). Evaluation and comparison of landslide susceptibility mapping methods: A case study for the Ulus district, Bartın, northern Turkey. Int. J. Geogr. Inf. Sci..

[28] Okalp K, Akgün H (2016). National level landslide susceptibility assessment of Turkey utilising public domain dataset. Environ. Earth Sci..

[29] Maes J (2017). Landslide risk reduction measures: A review of practices and challenges for the tropics. Prog. Phys. Geogr..

[30] Hong H (2019). Landslide susceptibility assessment at the Wuning area, China: A comparison between multi-criteria decision making, bivariate statistical and machine learning methods. Nat. Hazards.

[31] Pham BT, Prakash I (2019). A novel hybrid model of bagging-based naïve bayes trees for landslide susceptibility assessment. Bull. Eng. Geol. Env..

[32] Fang Z, Wang Y, Peng L, Hong H (2020). Integration of convolutional neural network and conventional machine learning classifiers for landslide susceptibility mapping. Comput. Geosci..

[33] Zêzere, J.-L. *et al*. Effects of landslide inventories uncertainty on landslide susceptibility modelling. In: *Landslide Processes: From Geomorphologic Mapping to Dynamic Modelling.Edition: Strasbourg*, 81–86 (2009).

[34] Chen W, Pourghasemi HR, Zhao Z (2017). A GIS-based comparative study of Dempster-Shafer, logistic regression and artificial neural network models for landslide susceptibility mapping. Geocarto Int..

[35] Aditian A, Kubota T, Shinohara Y (2018). Comparison of GIS-based landslide susceptibility models using frequency ratio, logistic regression, and artificial neural network in a tertiary region of Ambon, Indonesia. Geomorphology.

[36] Sevgen E, Kocaman S, Nefeslioglu HA, Gokceoglu C (2019). A novel performance assessment approach using photogrammetric techniques for landslide susceptibility mapping with logistic regression, ANN and random forest. Sensors.

[37] Sameen MI, Pradhan B, Lee S (2020). Application of convolutional neural networks featuring Bayesian optimisation for landslide susceptibility assessment. CATENA.

[38] Sun D, Wen H, Wang D, Xu J (2020). A random forest model of landslide susceptibility mapping based on hyperparameter optimization using Bayes algorithm. Geomorphology.

[39] LeCun Y, Bengio Y, Hinton G (2015). Deep learning. Nature.

[40] Chauhan S (2019). A comparison of shallow and deep learning methods for predicting cognitive performance of stroke patients from MRI lesion images. Front. Neuroinform..

[41] Aggarwal CC (2018). Neural Networks and Deep Learning.

[42] Wang Y, Fang Z, Hong H (2019). Comparison of convolutional neural networks for landslide susceptibility mapping in Yanshan County, China. Sci. Total Environ..

[43] Ding, A., Zhang, Q., Zhou, X. & Dai, B. in *2016 31st Youth Academic Annual Conference of Chinese Association of Automation (YAC).* 444–448 (IEEE, 2016).

[44] Xiao L, Zhang Y, Peng G (2018). Landslide susceptibility assessment using integrated deep learning algorithm along the China-Nepal highway. Sensors.

[45] Van Dao D (2020). A spatially explicit deep learning neural network model for the prediction of landslide susceptibility. CATENA.

[46] Huang F (2020). A deep learning algorithm using a fully connected sparse autoencoder neural network for landslide susceptibility prediction. Landslides.

[47] Bui DT, Tsangaratos P, Nguyen V-T, Van Liem N, Trinh PT (2020). Comparing the prediction performance of a Deep Learning Neural Network model with conventional machine learning models in landslide susceptibility assessment. CATENA.

[48] Cichy RM, Khosla A, Pantazis D, Torralba A, Oliva A (2016). Comparison of deep neural networks to spatio-temporal cortical dynamics of human visual object recognition reveals hierarchical correspondence. Sci. Rep..

[49] Prakash N, Manconi A, Loew S (2020). Mapping landslides on EO data: Performance of deep learning models vs traditional machine learning models. Remote Sens..

[50] Iran Meteorological Organization. http://www.irimo.ir (2021).

[51] Ghanbarian MA, Yassaghi A, Derakhshani R (2021). Detecting a sinistral transpressional deformation belt in the Zagros. Geosciences.

[52] Ghanbarian MA, Derakhshani R (2021). Systematic Variations in the Deformation Intensity in the Zagros Hinterland Fold-and-Thrust Belt.

[53] Aghanabati A (2004). Geology of Iran.

[54] Ghorbani, M. A summary of geology of Iran. In: *The Economic Geology of Iran*, 45–64 (Springer, 2013). 10.1007/978-94-007-5625-0_2.

[55] ArcGIS. (2021) https://desktop.arcgis.com/en/arcmap/10.4/get-started/setup/arcgis-desktop-quick-start-guide.htm.

[56] Reichenbach P, Rossi M, Malamud BD, Mihir M, Guzzetti F (2018). A review of statistically-based landslide susceptibility models. Earth Sci. Rev..

[57] Yao X, Tham L, Dai F (2008). Landslide susceptibility mapping based on support vector machine: A case study on natural slopes of Hong Kong, China. Geomorphology.

[58] Rossi M, Guzzetti F, Reichenbach P, Mondini AC, Peruccacci S (2010). Optimal landslide susceptibility zonation based on multiple forecasts. Geomorphology.

[59] Fox, J. *et al*. *Package ‘Car’* (R Foundation for Statistical Computing, 2018).

[60] Iran Water Resources Management Company. https://www.wrm.ir/ (2021).

[61] Rahman M, Chen N, Elbeltagi A, Islam MM, Alam M, Pourghasemi HR, Tao W, Zhang J, Shufeng T, Faiz H, Baig MA, Dewan A (2021). Application of stacking hybrid machine learning algorithms in delineating multi-type flooding in Bangladesh. J. Environ. Manage..

[62] Mersha T, Meten M (2020). GIS-based landslide susceptibility mapping and assessment using bivariate statistical methods in Simada area, northwestern Ethiopia. Geoenviron. Disasters.

[63] Ayalew L, Yamagishi H (2005). The application of GIS based logistic regression for landslide susceptibility mapping in the KakudaYahiko Mountains Central Japan. Geomorphology.

[64] Ahmad H, Ningsheng C, Rahman M, Islam MM, Pourghasemi HR, Hussain SF, Habumugisha JM, Liu E, Zheng H, Ni H, Dewan A (2021). Geohazards susceptibility assessment along the upper indus basin using four machine learning and statistical models. ISPRS Int. J. Geo Inf..

